# BEV-IP: Perioperative chemotherapy with bevacizumab in patients undergoing cytoreduction and intraperitoneal chemoperfusion for colorectal carcinomatosis

**DOI:** 10.1186/s12885-015-1954-x

**Published:** 2015-12-16

**Authors:** Wouter Willaert, Kurt Van Der Speeten, Gabriel Liberale, Wim Ceelen

**Affiliations:** Department of Surgical Oncology, Oost-Limburg Hospital, Genk, Belgium; Clinic of Digestive Surgical Oncology, Jules Bordet Institute, Brussels, Belgium; Department of Gastrointestinal Surgery, Ghent University Hospital, 2K12 IC UZ De Pintelaan 185, B-9000 Ghent, Belgium

**Keywords:** Bevacizumab, Colorectal cancer, Cytoreductive surgery, HIPEC, Perioperative chemotherapy, Peritoneal carcinomatosis

## Abstract

**Background:**

Selected patients with peritoneal carcinomatosis (PC) from colorectal cancer (CRC) benefit from cytoreductive surgery (CRS) combined with intraperitoneal chemoperfusion (IPC). However, even after optimal cytoreduction, systemic and locoregional recurrence are common. Perioperative chemotherapy with bevacizumab (BEV) may improve the outcome of these patients.

**Methods/Design:**

The BEV-IP study is a phase II, single-arm, open-label study aimed at patients with colorectal or appendiceal adenocarcinoma with synchronous or metachronous PC. This study evaluates whether perioperative chemotherapy including BEV in combination with CRS and oxaliplatin-based IPC results in acceptable morbidity and mortality (primary composite endpoint). Secondary endpoints are treatment completion rate, chemotherapy-related toxicity, pathological response, progression free survival, and overall survival.

**Discussion:**

The BEV-IP trial is the first prospective assessment of the safety and efficacy of perioperative chemotherapy combined with anti-angiogenic treatment in patients undergoing CRS and IPC for colorectal peritoneal metastases.

**Trial registration:**

ClinicalTrials.gov Identifier: NCT02399410 EudraCT number: 2015-001187-19 (registered March 9, 2015).

## Background

### Peritoneal carcinomatosis from colorectal cancer

Colorectal cancer (CRC) represents a major cause of cancer related mortality worldwide [1]. Over the past decades, advances in surgical management and identification of novel therapeutic targets have led to significant progress in the survival of patients with metastatic disease [2]. A notable exception, however, are patients with peritoneal carcinomatosis (PC) who are not only at risk of debilitating symptoms but in whom modern chemotherapy and targeted therapy are much less effective [3].

The epidemiology and risk factors for PC in CRC are not well established. In retrospective single centre series, the reported incidence of PC is approximately 7 % of patients at primary surgery, and 4 % to 19 % of patients during follow-up after curative surgery [4]. In a recent population-based cohort study from Stockholm County in Sweden, 4.8 % of 11,124 CRC patients had PC as the first and only site of metastatic disease [5]. Results from this cohort study as well as those from a large CRC cohort study in The Netherlands have identified several independent clinicopathological risk factors for synchronous PC: colon versus rectal cancer, right colon cancer, T stage, N stage, emergency and non-radical resection, younger age, and mucinous tumours [6]. Recent molecular research in a series of 524 CRC patients has indicated that those with BRAF mutant cancers (11 %) are at higher risk of PC (46 % vs 24 %, P = 0.001) [7].

### Systemic chemotherapy

When untreated, the outlook of patients with PC from CRC is grim. The French multicentre EVOCAPE 1 study found a median survival in patients with PC of 5.2 months [8]. The predictive and prognostic significance of PC in metastatic CRC (mCRC) patients treated with palliative chemotherapy with or without a targeted agent are difficult to assess since the presence of PC is usually not specified in the reported trials. There is a lack of high level evidence on systemic anticancer therapy in patients with mCRC limited to the peritoneal surfaces. Nevertheless, a number of observations can be made from the available literature. First, systemic chemotherapy prolongs survival in PC patients compared to best supportive care. In a series of 167 PC patients, Pelz et al. observed a median survival of 5 months in patients not receiving chemotherapy versus 11 and 12 months in patients receiving 5-fluorouracil (5-FU)/leucovorin (LV) and oxaliplatin (OX)/irinotecan (IRI)-based chemotherapy, respectively (*P* = 0.026 versus no chemotherapy) [9]. In the CAIRO 2 study, which randomized mCRC patients to either capecitabine, OX, and bevacizumab (BEV) or the same regimen plus cetuximab, the subgroup of patients with PC had a median survival of 15.2 months [10]. However, several authors have shown that the presence of PC represents an adverse predictive factor in patients treated with modern chemotherapy. Franko and colleagues analysed the outcome of PC versus other metastatic sites in mCRC patients included in the North Central Cancer Treatment Group trials N9741 (comparing FOLFIRI, FOLFOX, and IROX) and N9841(comparing IRI versus FOLFOX) [11]. They found that overall (OS) and progression free survival (PFS) were significantly worse in patients with PC (95 % confidence interval of the hazard ratio 1.2–1.5; *P* < 0.001 and 1.1–1.3; *P* = 0.001, respectively).

### Hyperthermic intraperitoneal chemoperfusion (HIPEC)

HIPEC was first described in an animal model in 1974 by Euler [12]. The first clinical application of combined cytoreductive surgery (CRS) and HIPEC was reported in 1980 by Spratt and colleagues, who treated a young patient suffering from pseudomyxoma peritonei with extensive surgery followed by intraperitoneal chemoperfusion (IPC) of Thiotepa under hyperthermic conditions using a delivery system consisting of a heat exchanger and pump [13]. After the procedure, the drains were left in place and 5 days later another HIPEC procedure with methotrexate was performed. In that publication, the authors stressed the importance of removing free floating cancer cells by the microfilters in the perfusion circuit. The advantage of intraoperative (as opposed to adjuvant) chemoperfusion is the possibility to achieve optimal chemotherapy exposure of all peritoneal surfaces at risk of peritoneal seeding. The use of hyperthermia is based on several observations. First, hyperthermia is selectively cytotoxic for malignant cells [14]. Second, the cytotoxicity of several chemotherapeutic agents such as the platinum compounds and the alkylating drugs is enhanced by hyperthermia [15]. Third, hyperthermia enhances tissue perfusion and oxygenation and may improve drug penetration. Los and colleagues demonstrated a significant increase in peritoneal tumour platinum concentrations when intraperitoneal (IP) cisplatin (CIS) therapy was combined with regional hyperthermia (41.5 °C) in a rat colon cancer model [16].

Table 1 summarizes the most important clinical studies in CRC published so far. Only one randomized prospective trial has evaluated palliative treatment versus surgery and HIPEC in PC from CRC. Verwaal and co-authors reported a randomized controlled trial comparing systemic 5FU/LV and palliative surgery when required versus extensive CRS and HIPEC using mitomycin C (35 mg/m^2^) followed by systemic 5FU/LV in patients with PC from CRC [17, 18]. The median disease specific survival was 12.6 months in the control arm and 22.2 months in the CRS with HIPEC arm (*P* = 0.028); survival was significantly better in patients with no more than five of seven abdominal regions affected and in patients in whom a macroscopically complete resection was achieved. The significance of this trial is somewhat limited by the use of systemic chemotherapy that is no longer regarded as the standard of care in this setting and by the fact that the question whether extensive CRS in itself (without added HIPEC) would achieve a similar outcome remains unanswered. The results from a multinational retrospective analysis of 506 patients with PC from CRC treated with CRS and HIPEC showed a median OS of 19 months; patients in whom a complete cytoreduction was achieved had a median OS of 32.4 months [19]. Multivariate analysis revealed that other variables associated with survival gain were treatment by a second procedure, limited disease extent, age < 65 years and use of adjuvant chemotherapy. A systematic review performed by Yan et al. demonstrated that with complete cytoreduction a median OS from 28 to 60 months and 5-year OS ranging from 22 to 49 % may be reached [20]. Similar results were obtained in a Belgian and an Italian multicentre registry [21, 22]. Overall, the quality of the available evidence is low, but it is reasonable to assume that in selected patients in whom complete cytoreduction can be achieved a significant prolongation of survival is possible.

**Table 1:** Clinical studies of HIPEC in patients with peritoneal carcinomatosis from colorectal origin

Author	N	IP chemo (mg/m^2^)	Mb (%)	Mt (%)	RR (%)	HS (d)	MS (m)	prognostic variables in multivariate analysis
CRT								
Verwaal [16, 17]	49^a^	MMC 35	-	8	-	29	22.3	>5/7 regions affected; CC
Multicentre								
Elias [22]	523^b^	MMC 30–50, CIS 50–100 OX 360–460, IRN 200	31	3.3	-	18	30.1	PCI, CC, nodal status, adjuvant chemotherapy
Glehen [18]	506^c^	MMC, CIS, OX	23	4	10.7	-	19.2	PCI, CC, adjuvant chemotherapy, age <65y
Hompes [20]	48	OX 460	52	0	21	20	NR	-
Cavaliere [21]	146	CIS 25, OX 460, MMC 33	27	2.7	-	20	21	CC, liver metastasis
Quenet [23]	146	OX 300–460, IRN 200	47	4	-	-	41	PCI, nodal status
Monocentric								
Franko [24]	67	MMC 40	-	-	-	-	35	-
Cashin [25]	69	MMC 30, OX 460, IRN 360	40.6	4.3	-	-	34	-
Shen [26]	77	MMC 30-40	30	12	-	10	16	CC, bowel obstruction, ascites
Vaira [27]	40	CIS 100, MMC 16–35, OX 460	55	2.5	23	-	-	-
Ceelen [28]	166	OX 200–460, MMC 35	35	2.4	-	-	27	CC, neoadjuvant therapy with BEV

*BEV* bevacizumab, *CC* completeness of cytoreduction, *CIS* cisplatin, *HIPEC* hyperthermic intraperitoneal chemoperfusion, *HS* hospital stay, *IP* intraperitoneal, *IRN* irinotecan; *Mb* postoperative morbidity, *MMC* mitomycin C; *MS* median survival in months, *Mt* postoperative mortality, *NR* not reached, *OX* oxaliplatin, *PCI* peritoneal cancer index, *RR* reoperation rate; ^a^includes 13 % appendix cancer; ^b^18 % early postoperative intraperitoneal chemotherapy (EPIC) alone; ^c^24 % EPIC alone

### VEGF, Bevacizumab, and peritoneal carcinomatosis

The vascular endothelial growth factor A (VEGF-A) plays a central role in tumour associated angiogenesis and in the pathogenesis of malignant ascites [23]. BEV (Avastin®, Genentech/Roche) is a humanized monoclonal antibody against circulating VEGF-A. The potential role of inhibiting VEGF using BEV in patients with PC from CRC is based on several theoretical considerations. First, the combination of BEV with doublet (combination) chemotherapy is considered an active first line strategy in patients with mCRC [24]. Since many patients with isolated PC from CRC harbour undetected systemic disease and will eventually recur, the addition of potent systemic therapy to a locoregional approach such as CRS with HIPEC is warranted. Second, BEV is known to result in the lowering of the tumour’s interstitial fluid pressure by vascular normalization, which may result in enhanced delivery of IP chemotherapy [25–27]. Third, preclinical and early clinical data demonstrate a role for BEV in the treatment of PC and/or ascites from CRC or ovarian cancer [28–31]. In a recent clinical study, Passot and colleagues determined intravenous (IV) and IP VEGF levels before and after CRS and HIPEC [32]. They found that the IP VEGF concentration increased significantly after surgery and that neoadjuvant BEV was associated with a lower IP VEGF level in a multivariate model. Finally, neoadjuvant combination therapy with BEV may result in downstaging of disease extent, possibly leading to improved outcome. In colorectal liver metastases, neoadjuvant BEV combined with OX-based chemotherapy was shown to result in enhanced pathological response and improved PFS and OS [33]. Recent data from Ghent University Hospital analysing the survival rate of a cohort of 166 CRC patients treated with CRS and HIPEC showed that neoadjuvant combination chemotherapy with BEV was significantly associated with better OS in multivariate analysis [34]. In addition, we recently demonstrated that pretreatment with BEV leads to a markedly reduced IFP in a mouse HT29 CRC model of IPC with OX, which may allow for deeper penetration and higher tumour drug concentration [35].

A potential drawback of perioperative BEV is the risk of increased surgical morbidity. In a French multicentre retrospective study, the addition of BEV to neoadjuvant chemotherapy resulted in a significantly increased rate of major morbidity (34 vs. 19 %, P = 0.020) [36]. There were no differences in postoperative mortality or anastomotic leak rate. Also, both groups were significantly different in terms of associated liver resection (twice more common in the BEV group) and chemoperfusion drug regimen. In general, however, perioperative use of BEV is considered relatively safe. A recent review suggested that the overall risk of serious BEV related adverse events in surgical patients is very low, provided that a time frame of six weeks is respected [37].

The aim of the BEV-IP trial is to test the hypothesis that perioperative chemotherapy combined with BEV results in acceptable morbidity and mortality.

## Methods/Design

### Study design

The BEV-IP study is a phase II, single-arm, open-label study. The study was initiated by the Department of Gastrointestinal Surgery of the Ghent University Hospital in collaboration with the Department of Surgical Oncology of the Oost-Limburg Hospital in Genk, and the Clinic of Digestive Surgical Oncology of Jules Bordet Institute in Brussels. The trial will, however, be open to participation by additional centers.

### Study objectives and endpoints

The objective of the BEV-IP study is to assess the safety and efficacy of perioperative combination chemotherapy with BEV in colorectal PC patients treated with CRS and IPC. The primary (composite) endpoint is three month surgical morbidity and mortality, calculated using the Dindo-Clavien classification [38]. Specifically, the endpoint will be considered reached whenever a grade IIIb or higher grade complication is reached. Secondary endpoints include other (minor) morbidity, chemotherapy related toxicity, treatment completion rate, pathological response, PFS (defined as the time interval between start of protocol treatment and disease progression or death), and OS (calculated from start of protocol treatment until death). In addition, translational research will be performed on blood, serum, peritoneal fluid, and tumor tissue samples obtained at various time points.

### Inclusion criteria

Eligible patients suffer from synchronous or metachronous biopsy proven peritoneal metastases from colorectal origin. The following inclusion criteria should be met: age over 18 years, Karnofsky index > 70 %, adequate mental faculty allowing to understand the proposed treatment protocol and provide informed consent, estimated life expectancy > 6 months, absence of any other concurrent malignant disease, serum creatinine ≤ 1.5 mg/dl or a calculated glomerular filtration rate ≥ 60 mL/min/1.73 m^2^, serum total bilirubin ≤ 1.5 mg/dl except for known Gilbert’s disease, platelet count > 100,000/μl, hemoglobin > 9 g/dl, neutrophil granulocytes > 1,500/ml, International Normalized Ratio ≤ 2, absence of alcohol and/or drug abuse, no inclusion in other clinical trials interfering with the study protocol, no concurrent chronic systemic immune therapy, chemotherapy or hormone therapy not indicated in the study protocol, absence of any severe organ insufficiency, no pregnancy or breast feeding, adequate contraception in fertile patients and written informed consent. In addition, the disease should be deemed optimally resectable (CC-0 or CC-1) with preservation of an adequate quality of life. The estimated peritoneal cancer index should ideally not exceed 25, but the location and distribution of peritoneal spread are often more important than the size and number of implants.

Patients with severe or uncontrolled cardiac pathology, including recent (<6 months) occurrence of myocardial infarction, the presence of congestive cardiac failure, of symptomatic angor pectoris despite optimal medical care, of cardiac arrhythmia requiring medical treatment with insufficient rhythm control, or uncontrolled arterial hypertension are excluded. Further exclusion criteria are active bacterial, viral or fungal infection, active gastro-duodenal ulcer, parenchymal liver disease (any stage cirrhosis), uncontrolled diabetes mellitus, severe obstructive or restrictive respiratory insufficiency, psychiatric pathology capable of affecting comprehension and judgment faculty, tumor in the presence of obstruction, evidence of extra-abdominal disease or extensive liver metastasis, and known allergy to any of the trial related drugs.

### Interventions

**Fig. 1:**
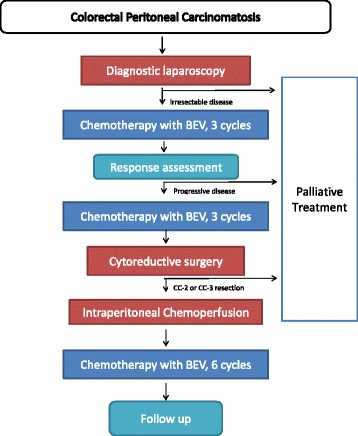
Flowchart of the BEV-IP study

Figure 1 illustrates the flowchart of all trial related procedures.

#### Staging procedures

Clinical staging will include at least CT scan of the chest and abdomen with itravenous (IV) and oral contrast and measurement of carcinoembryonic antigen (CEA). Additional imaging such as ^18^F-FDG-PET-CT, MRI, or diffusion weighted (DWI) - MRI are optional. Peritoneal disease extent is scored according to Sugarbaker’s peritoneal cancer index (PCI, score 0–39) [39]. Patients meeting the inclusion criteria are planned for diagnostic laparoscopy, which is recommended in all patients. The aim of laparoscopy is to obtain tumor tissue for diagnostic confirmation and for translational research. In addition, it may confirm resectability by allowing inspection of the peritoneal surfaces, specifically at the small bowel surface and mesentery. Alternatively, a tissue diagnosis may be obtained by transluminal or transparietal punction biopsy.

#### Neoadjuvant therapy

Neoadjuvant therapy consists of a combination of 5-fluorouracil or capecitabine, oxaliplatin or irinotecan, and bevacizumab for a total of 6 biweekly administrations. The exact drug dose and schedule is according to local practice, but typically includes oxaliplatin at a dose of 85–100 mg/m^2^, irinotecan 180 mg/m^2^, and BEV 5 mg/kg. In patients with extensive abdominal wall metastatic implants, radiotherapy may be considered in addition to chemotherapy.

#### Response assessment

Therapy response is assessed after 3 therapy cycles (6 weeks of treatment) by clinical examination, CEA measurement, and CT scan of the chest and abdomen. Other imaging modalities are optional. Patients with clinical signs or symptoms of disease progression (e.g. malignant ascites, ureteral stenosis or bowel obstruction) are excluded. In patients with measurable peritoneal lesions, response will be graded according to the RECIST 1.1 criteria: (1) complete response (CR), (2) partial response (PR, at least 30 % decrease in the sum of diameters of target lesions), (3) stable disease (SD) and (4) progressive disease (PD, new lesions or at least 20 % increase in the sum of diameters of target lesions) [40]. Patients with response or stable disease are continued on the same treatment until 6 cycles. Upon completion of the neoadjuvant treatment schedule, a second response assessment is perfomed using biochemistry, imaging, and clinical assessment. Toxicity from neoadjuvant therapy will be recorded and scored using the Common Terminology Criteria for Adverse Events (CTCAE) version 4.0 [41]. In responding patients, surgery and IPC are planned within 6–8 weeks completion of neoadjuvant therapy. A minimal interval of 6 weeks between the last dose of BEV and surgery will be respected.

#### Surgery and chemoperfusion

Peritoneal ascites or samples from peritoneal lavage with saline are obtained for VEGF analysis before and after CRS. The abdomen is explored systematically and CRS proceeds if all disease is deemed resectable and a PCI ≤ 25 is confirmed. Tumor tissue samples (2×5 mm^3^) are obtained and snap frozen for translational research. The result of CRS will be scored according to the completeness of cytoreduction (CC) score: CC-0, no visible residual tumour; CC-1, residual tumour nodules ≤ 2.5 mm in diameter; CC-2: residual tumor nodules between 2.5 mm and 2.5 cm, and CC-3, residual disease > 2.5 cm in size [42]. Intraperitoneal chemoperfusion is performed according to local preference; the open method (coliseum) is preferred. Oxaliplatin (200–460 mg/m^2^) in dextrose 5 % (2 liter/m^2^) is administered IP immediately following 5-FU 400 mg/m^2^ IV and LV 20 mg/m^2^ IV. Perfusion temperature and duration of IPC are according to local preference. Postoperative complications are recorded during a period of three months postoperatively and scored according to the Dindo-Clavien classification.

#### Pathology reporting

Specific attention will be paid to analysis of preoperative chemotherapy response of the primary and/or the peritoneal metastases. Tumor regression will be scored according to the tumor regression grade (TRG) as described by Rubbia-Brandt: TRG1, absence of tumor cells replaced by abundant fibrosis; TRG2, rare scattered residual tumor cells and abundant fibrosis; TRG3, a large amount of residual tumor cells with predominant fibrosis; TRG4, tumor cells predominating over fibrosis; and TRG5, almost exclusively tumor cells without fibrosis [43]. In addition, the mean percentages of necrosis and fibrosis are assessed separately according to published methods [44]. In patients with synchronous PC, the primary tumor will be staged according to the 7^th^ edition of the UICC/AJCC TNM staging system.

#### Follow-up

Within 4–6 weeks postoperatively, baseline measurement of CEA and a CT scan of the chest and abdomen are performed. Adjuvant chemotherapy is initiated from 6–8 weeks postoperatively, with a maximum interval since the time of surgery of three months. Six additional cycles of adjuvant chemotherapy are administered, identical to the neoadjuvant therapy. Reassessment with CEA measurement and CT scan of the chest and abdomen are performed after completion of the adjuvant chemotherapy course. Further assessments using biochemistry, imaging, and clinical evaluation are planned 3, 6, 9, 12, 18, and 24 months after completion of adjuvant chemotherapy. Progressive patients are managed according to local standard of care.

#### Patient reported outcomes

Health related quality of life (HRQOL) is an important endpoint in trials investigating novel therapies in patients with metastatic cancer. The EORTC QLQ C-30 and SF-36 questionnaires will be taken at different time points: after inclusion (but before start of neoadjuvant therapy), the day before surgery, and at postoperative month 3, 6, 12, 18, and 24. The EORTC questionnaires will be scored according to the scoring manual (EORTC, third edition, 2001). The 36-Item Short Form Health Survey (SF-36, RAND health) will be scored according to the instructions from RAND Health (2009 version).

#### Translational research

Translational research will be aimed at identifying biomarkers that predict response and toxicity to BEV containing chemotherapy. Standard histology and immunohistochemistry is used to analyze microvessel density and maturation as well as protein expression of VEGF-A and HIF-1. Molecular biology techniques will include gene expression of VEGF, VEGFR1, TSP-2, and EGFR, mutation status of KRAS/NRAS/BRAF, and germline polymorphisms (SNP’s) in VEGF dependent angiogenesis pathways. In addition, circulating (serum) biomarkers will be measured including VEGF, soluble VEGFR-2, VEGF-C, VEGF-D, PlGF, bFGF, HGF, SDF-1, MCP-3, Ang-2, IL-6, and IL-8. In peritoneal fluid samples, peritoneal VEGF concentrations are measured. In addition, we will analyze circulating tumor cells, circulating endothelial cells, circulating endothelial progenitor cells, and circulating exosomes.

### Statistical considerations

The sample size (n = 45) was calculated to allow the estimation of the primary endpoint with sufficiently narrow confidence intervals to inform further development. Three month morbidity and mortality rates reported from studies or reviews of similar approaches but without BEV, ranged from 23 to 31 %, and from 3 to 4 %, respectively. Based on the literature, we expect to observe a morbidity rate of 27 % and a mortality rate of 3.5 %. Using effect methods, the proposed sample size will provide 95 % confidence intervals of about 14 %–40 % and 0.8 %–14 % for morbidity and mortality, respectively. In general, morbidity rates as well as the fraction of operated patients who receive postoperative chemotherapy will be estimated to ± 14 % or better. A total of 60 patients will be included in order to account for the estimated drop-out rate of 33 %. Patients will be followed up for 24 months postoperatively. Anonymized individual patient data will be extracted from the medical record onto paper case report forms (CRF). The local investigator will also report all adverse events in the source documents and CRFs. The serious adverse events will be reported within time periods specified in the protocol. Given the uncertainty of potential operative complications caused by BEV, an interim analysis is planned after each additional cohort of 10 patients is included in the study. The study will be terminated when the observed major morbidity rate is >40 % and/or in hospital mortality is >14 % at any given point in time.

### Ethical considerations

The study will be conducted in agreement with either the Declaration of Helsinki (Tokyo, Venice, Hong Kong, Somerset West and Edinburgh amendments) or the laws and regulations of the country, whichever provides the greatest protection of the patient. The protocol was written, and the study will be conducted according to the international conference on harmonization guidelines for Good Clinical Practice (ICH-GCP). The protocol was approved on May 13, 2015 by the Ethical Committee of Ghent University Hospital. All data collected on a patient’s health for the purpose of research will be kept confidential. The patient’s identity will never be disclosed.

## Discussion

The concept of cytoreductive surgery and intraperitoneal chemoperfusion has significantly improved the outlook of patients with pseudomyxoma peritonei originating from mucinous appendiceal neoplasms [45]. It was therefore rational to expand the use of CRS and IPC to PC from colorectal cancer, notwithstanding the less favourable disease biology. Although significant prolongation of PFS may be achieved in selected patients, recurrence after CRS and IPC is common. Braam and coworkers reported that, even after complete cytoreduction and HIPEC, 73 % of all patients recurred [46]. Isolated peritoneal recurrence was noted in 43 % of these patients while 57 % developed systemic disease with or without peritoneal recurrence. At the same time, systemic therapy for metastatic CRC has improved considerably over the past decades. Even though the response of peritoneal metastases is often less pronounced compared to solid organ metastases, there is little doubt that modern chemotherapy is active against PC from colorectal origin. Kerscher et al. noted that, over the past two decades, the OS of PC patients has improved from 7 to 18 months concurrent with increasing use of active chemotherapy regimens [47]. In a retrospective analysis of 115 PC patients treated with preoperative irinotecan or oxaliplatin based chemotherapy followed by CRS and IPC, Passot and coworkers demonstrated a complete and major pathological response rate of the peritoneal disease in 10 % and 20 % of patients respectively [48]. In addition, they demonstrated that among the various chemotherapy lines used, FOLFOX with BEV resulted in the best OS, a finding which was also reported by our own group [49]. Given the high risk of recurrence and the expected activity against peritoneal disease, perioperative chemotherapy with BEV may result in a benefit similar to the setting of colorectal liver metastases [50]. The drawback of neoadjuvant chemotherapy is that patients who do not respond may progress and eventually become unresectable. However, it may be argued that this scenario points to an unfavourable disease biology in patients who would not benefit from extensive surgery anyway, but would better be treated with alternative systemic treatment. In addition, the reported rates of serious BEV-related postoperative adverse events are low, if a time to surgery of 5–6 weeks is respected [37]. The addition of BEV to first- or second-line 5-FU-based chemotherapy increases PFS and OS [24]. Moreover, neoadjuvant combination therapy with BEV improves regression of CRC liver metastases and lowers the IP VEGF level [33]. Intraperitoneal administration of BEV has some activity in the palliation of malignant ascites [28–30]. Furthermore, research in peritoneal metastatic mice models shows that BEV inhibits the growth of peritoneal nodules and ascites [31]. Hence, an intensified perioperative therapy with doublet chemotherapy plus BEV may downstage the disease before performing CRS, limit the extensiveness of surgery, increase the rates of CC-0/1 resection, and improve control of locoregional and distant recurrence.

The BEV-IP trial is the first to investigate the safety and efficacy of perioperative combination chemotherapy with BEV in combination with CRS and IPC with OX in patients with synchronous or metachronous peritoneal carcinomatosis from CRC. The results will help to define the place of systemic chemotherapy combined with anti-angiogenic treatment in the perioperative setting in patients with PC from colorectal orgin who are candidates for CRS and IPC.

## Abbreviations

5-FU: 5-fluorouracil
BEV: bevacizumab
CEA: carcinoembryonic antigen
CIS: cisplatin
CR: complete response
CRC: colorectal cancer
CRF: case report form
CRS: cytoreductive surgery
CT: computed tomography
FOLFIRI: folinic acid + fluorouracil + irinotecan
FOLFOX: folinic acid + fluorouracil + oxaliplatin
HIPEC: hyperthermic intraperitoneal chemoperfusion
IP: Intraperitoneal
IPC: intraperitoneal chemoperfusion
IRI: irinotecan
IROX: irinotecan + oxaliplatin
IV: intravenous
LV: leucovorin
mCRC: metastatic colorectal cancer
MRI: magnetic resonance imaging
NCI-CTCAE: common terminology criteria for adverse events
OS: overall survival
PC: peritoneal carcinomatosis
PD: progressive disease
PET: positron emission tomography
PFS: progression free survival
PM: peritoneal metastases
PR: partial response
SD: stable disease

## References

[1] Herszenyi L, Tulassay Z (2010). Epidemiology of gastrointestinal and liver tumors. Eur Rev Med Pharmacol Sci.

[2] Peeters M, Price T (2012). Biologic therapies in the metastatic colorectal cancer treatment continuum - Applying current evidence to clinical practice. Cancer Treat Rev.

[3] Ceelen WP. Current management of peritoneal carcinomatosis from colorectal cancer. Minerva Chir.2013;68(1):77–86. 23584267

[4] Koppe MJ, Boerman OC, Oyen WJG, Bleichrodt RP (2006). Peritoneal carcinomatosis of colorectal origin - Incidence and current treatment strategies. Ann Surg.

[5] Segelman J, Granath F, Holm T, Machado M, Mahteme H, Martling A (2012). Incidence, prevalence and risk factors for peritoneal carcinomatosis from colorectal cancer. Br J Surgery.

[6] Lemmens VE, Klaver YL, Verwaal VJ, Rutten HJ, Coebergh JWW, de Hingh IH (2011). Predictors and survival of synchronous peritoneal carcinomatosis of colorectal origin: a population-based study. Int J Cancer.

[7] Tran B, Kopetz S, Tie J, Gibbs P, Jiang Z-Q, Lieu CH (2011). Impact of BRAF Mutation and Microsatellite Instability on the Pattern of Metastatic Spread and Prognosis in Metastatic Colorectal Cancer. Cancer.

[8] Sadeghi B, Arvieux C, Glehen O, Beaujard AC, Rivoire M, Baulieux J (2000). Peritoneal carcinomatosis from non-gynecologic malignancies - Results of the EVOCAPE 1 multicentric prospective study. Cancer.

[9] Pelz JOW, Chua TC, Esquivel J, Stojadinovic A, Doerfer J, Morris DL, et al. Evaluation of Best Supportive Care and Systemic Chemotherapy as Treatment Stratified according to the retrospective Peritoneal Surface Disease Severity Score (PSDSS) for Peritoneal Carcinomatosis of Colorectal Origin. BMC Cancer. 2010;10. 10.1186/1471-2407-10-689 PMC3014907 21176206

[10] Klaver YL, Simkens LH, Lemmens VE, Koopman M, Teerenstra S, Bleichrodt RP (2012). Outcomes of colorectal cancer patients with peritoneal carcinomatosis treated with chemotherapy with and without targeted therapy. Eur J Surg Oncol.

[11] Franko J, Shi Q, Goldman CD, Pockaj BA, Nelson GD, Goldberg RM (2012). Treatment of Colorectal Peritoneal Carcinomatosis With Systemic Chemotherapy: A Pooled Analysis of North Central Cancer Treatment Group Phase III Trials N9741 and N9841. J Clin Oncol.

[12] Euler J, Prieschi A, Wenzl J, Sauerman G, Klockler K, Kretschm G (1974). Hyperthermic peritoneal perfusion in ascites tumors in rats. Wien Klin Wochenschr.

[13] Spratt JS, Adcock RA, Muskovin M, Sherrill W, Mckeown J (1980). Clinical Delivery System for Intra-Peritoneal Hyperthermic Chemotherapy. Cancer Res.

[14] Hildebrandt B, Wust P, Ahlers O, Dieing A, Sreenivasa G, Kerner T (2002). The cellular and molecular basis of hyperthermia. Critical Reviews in Oncology Hematology.

[15] Raaphorst GP, Yang DP (2005). The evaluation of thermal cisplatin sensitization in normal and XP human cells using mild hyperthermia at 40 and 41 degrees C. Anticancer Res.

[16] Los G, Sminia P, Wondergem J, Mutsaers PHA, Havemen J, Huinink DT (1991). Optimization of Intraperitoneal Cisplatin Therapy with Regional Hyperthermia in Rats. Eur J Cancer.

[17] Verwaal VJ, van Ruth S, de Bree E, van Slooten GW, van Tinteren H, Boot H (2003). Randomized trial of cytoreduction and hyperthermic intraperitoneal chemotherapy versus systemic chemotherapy and palliative surgery in patients with peritoneal carcinomatosis of colorectal cancer. J Clin Oncol.

[18] Verwaal VJ, Bruin S, Boot H, van Slooten G, van Tinteren H (2008). 8-year follow-up of randomized trial: Cytoreduction and hyperthermic intraperitoneal chemotherapy versus systemic chemotherapy in patients with peritoneal carcinomatosis of colorectal cancer. Ann Surg Oncol.

[19] Glehen O, Kwiatkowski F, Sugarbaker PH, Elias D, Levine EA, De Simone M (2004). Cytoreductive surgery combined with perioperative intraperitoneal chemotherapy for the management of peritoneal carcinomatosis from colorectal cancer: A multi-institutional study. J Clin Oncol.

[20] Yan TD, Black D, Savady R, Sugarbaker PH (2006). Systematic review on the efficacy of cytoreductive surgery combined with perioperative intraperitoneal chemotherapy for peritoneal carcinomatosis from colorectal carcinoma. J Clin Oncol.

[21] Hompes D, D'Hoore A, Van Cutsem E, Fieuws S, Ceelen W, Peeters M (2012). The Treatment of Peritoneal Carcinomatosis of Colorectal Cancer with Complete Cytoreductive Surgery and Hyperthermic Intraperitoneal Peroperative Chemotherapy (HIPEC) with Oxaliplatin: A Belgian Multicentre Prospective Phase II Clinical Study. Ann Surg Oncol.

[22] Cavaliere F, De Simone M, Virzi S, Deraco M, Rossi CR, Garofalo A (2011). Prognostic factors and oncologic outcome in 146 patients with colorectal peritoneal carcinomatosis treated with cytoreductive surgery combined with hyperthermic intraperitoneal chemotherapy: Italian multicenter study S.I.T.I.L.O. EJSO.

[23] Tamsma JT, Keizer HJ, Meinders AE (2001). Pathogenesis of malignant ascites: Starling's law of capillary hemodynamics revisited. Ann Oncol.

[24] Welch S, Spithoff K, Rumble RB, Maroun J (2010). Gastrointestinal Canc Dis Site G: Bevacizumab combined with chemotherapy for patients with advanced colorectal cancer: a systematic review. Ann Oncol.

[25] Olsen MWB, Ley CD, Junker N, Hansen AJ, Lund EL, Kristjansen PEG (2006). Angiopoietin-4 inhibits angiogenesis and reduces interstitial fluid pressure. Neoplasia.

[26] Nakahara T, Norberg SM, Shalinsky DR, Hu-Lowe DD, McDonald DM (2006). Effect of inhibition of vascular endothelial growth factor signaling on distribution of extravasated antibodies in tumors. Cancer Res.

[27] Willett CG, Boucher Y, di Tomaso E, Duda DG, Munn LL, Tong RT (2004). Direct evidence that the VEGF-specific antibody bevacizumab has antivascular effects in human rectal cancer. Nat Med.

[28] Kobold S, Hegewisch-Becker S, Oechsle K, Jordan K, Bokemeyer C, Atanackovic D (2009). Intraperitoneal VEGF Inhibition Using Bevacizumab: A Potential Approach for the Symptomatic Treatment of Malignant Ascites?. Oncologist.

[29] Hamilton CA, Maxwell GL, Chemofsky MR, Bernstein SA, Farley JH, Rose GS (2008). Intraperitoneal bevacizumab for the palliation of malignant ascites in refractory ovarian cancer. Gynecol Oncol.

[30] Bellati F, Napoletano C, Ruscito I, Pastore M, Pernice M, Antonilli M (2010). Complete remission of ovarian cancer induced intractable malignant ascites with intraperitoneal bevacizumab. Immunological observations and a literature review. Invest New Drugs.

[31] Yagi Y, Fushida S, Harada S, Tsukada T, Kinoshita J, Oyama K (2010). Biodistribution of humanized anti-VEGF monoclonal antibody/bevacizumab on peritoneal metastatic models with subcutaneous xenograft of gastric cancer in mice. Cancer Chemother Pharmacol.

[32] Passot G, Bakrin N, Garnier L, Roux A, Vaudoyer D, Wallet F (2014). Intraperitoneal vascular endothelial growth factor burden in peritoneal surface malignancies treated with curative intent: The first step before intraperitoneal anti-vascular endothelial growth factor treatment?. Eur J Cancer.

[33] Klinger M, Tamandl D, Eipeldauer S, Hacker S, Herberger B, Kaczirek K (2010). Bevacizumab Improves Pathological Response of Colorectal Cancer Liver Metastases Treated with XELOX/FOLFOX. Ann Surg Oncol.

[34] Ceelen W, Van Nieuwenhove Y, Vande Putte D, Pattyn P (2014). Neoadjuvant Chemotherapy with Bevacizumab May Improve Outcome after Cytoreduction and Hyperthermic Intraperitoneal Chemoperfusion (HIPEC) for Colorectal Carcinomatosis. Ann Surg Oncol.

[35] Gremonprez F, Descamps B, Izmer A, Vanhove C, Vanhaecke F, De Wever O, et al. Pretreatment with VEGF(R)-inhibitors reduces interstitial fluid pressure, increases intraperitoneal chemotherapy drug penetration, and impedes tumor growth in a mouse colorectal carcinomatosis model. Oncotarget. 2015 Oct 6;6(30):29889-900. 10.18632/oncotarget.5092 PMC4745770 26375674

[36] Eveno C, Passot G, Goere D, Soyer P, Gayat E, Glehen O (2014). Bevacizumab Doubles the Early Postoperative Complication Rate after Cytoreductive Surgery with Hyperthermic Intraperitoneal Chemotherapy (HIPEC) for Peritoneal Carcinomatosis of Colorectal Origin. Ann Surg Oncol.

[37] Hompes D, Ruers T (2011). Review: Incidence and clinical significance of Bevacizumab-related non-surgical and surgical serious adverse events in metastatic colorectal cancer. EJSO.

[38] Dindo D, Demartines N, Clavien PA (2004). Classification of surgical complications - A new proposal with evaluation in a cohort of 6336 patients and results of a survey. Ann Surg.

[39] Sugarbaker PH (2005). Peritoneal surface oncology: review of a personal experience with colorectal and appendiceal malignancy. Tech Coloproctol.

[40] Schwartz LH, Bogaerts J, Ford R, Shankar L, Therasse P, Gwyther S (2009). Evaluation of lymph nodes with RECIST 1.1. Eur J Cancer.

[41] Younan R, Kusamura S, Baratti D, Cloutier A-S, Deraco M (2008). Morbidity, toxicity, and mortality classification systems in the local regional treatment of peritoneal surface malignancy. J Surg Oncol.

[42] Glehen O, Gilly FN (2003). Quantitative prognostic indicators of peritoneal surface malignancy: carcinomatosis, sarcomatosis, and peritoneal mesothelioma. Surg Oncol Clin N Am.

[43] Rubbia-Brandt L, Giostra E, Brezault C, Roth AD, Andres A, Audard V (2007). Importance of histological tumor response assessment in predicting the outcome in patients with colorectal liver metastases treated with neo-adjuvant chemotherapy followed by liver surgery. Ann Oncol.

[44] Loupakis F, Schirripa M, Caparello C, Funel N, Pollina L, Vasile E (2013). Histopathologic evaluation of liver metastases from colorectal cancer in patients treated with FOLFOXIRI plus bevacizumab. Br J Cancer.

[45] Chua TC, Moran BJ, Sugarbaker PH, Levine EA, Glehen O, Gilly FN (2012). Early- and long-term outcome data of patients with pseudomyxoma peritonei from appendiceal origin treated by a strategy of cytoreductive surgery and hyperthermic intraperitoneal chemotherapy. J Clin Oncol.

[46] Braam HJ, van Oudheusden TR, de Hingh IH, Nienhuijs SW, Boerma D, Wiezer MJ, et al. Patterns of recurrence following complete cytoreductive surgery and hyperthermic intraperitoneal chemotherapy in patients with peritoneal carcinomatosis of colorectal cancer. J Surg Oncol. 2014. 10.1002/jso.23597 24619813

[47] Kerscher AG, Chua TC, Gasser M, Maeder U, Kunzmann V, Isbert C (2013). Impact of peritoneal carcinomatosis in the disease history of colorectal cancer management: a longitudinal experience of 2406 patients over two decades. Br J Cancer.

[48] Passot G, You B, Boschetti G, Fontaine J, Isaac S, Decullier E (2014). Pathological Response to Neoadjuvant Chemotherapy: A New Prognosis Tool for the Curative Management of Peritoneal Colorectal Carcinomatosis. Ann Surg Oncol.

[49] Gremonprez F, Willaert W, Ceelen W. Intraperitoneal chemotherapy (IPC) for peritoneal carcinomatosis: review of animal models. J Surg Oncol. 2014 Feb;109(2):110-6. 10.1002/jso.23464 24122416

[50] Wong R, Cunningham D, Barbachano Y, Saffery C, Valle J, Hickish T (2011). A multicentre study of capecitabine, oxaliplatin plus bevacizumab as perioperative treatment of patients with poor-risk colorectal liver-only metastases not selected for upfront resection. Ann Oncol.

